# Spike history model for neural control

**DOI:** 10.1186/1471-2202-15-S1-P96

**Published:** 2014-07-21

**Authors:** T Kameneva, M Abramian, DB Grayden, AN Burkitt, H Meffin

**Affiliations:** 1 NeuroEngineering Laboratory, Department of Electrical Electronic Engineering, The University of Melbourne, Australia; 2 Centre for Neural Engineering, The University of Melbourne, Australia; 3 National ICT Australia, Victoria Research Lab, Australia; 4 Graduate School of Biomedical Engineering, The University of New South Wales, Australia; 5 Bionics Institute, East Melbourne, Australia

## 

There is increased interest from the research community and clinicians to implement closed-loop stimulation strategies in neurobionic devices. That is, to adjust stimulation levels dynamically based on the responses of neural tissue in real time. To adjust electrical stimulation in a closed-loop bionic device, a model-based controller design can be implemented. Here, we collect experimental data from retina slices and use data-driven technique to model neural dynamics. Our motivation comes from visual prostheses.

*In vitro* experiments were conducted on NZ white rabbit retina tissue. Electrical stimulation of the retinal ganglion cells consisted of a train of pulses whose amplitudes had a white Gaussian distribution with zero mean and standard deviation of 1uA. In this pulse train, there were a total of 5000 biphasic pulses with 100µs/phase duration, equal phase amplitude, and no interphase delay. Frequency pulse trains of 25-1000Hz were used.

Experimental data were used to characterize the response properties of neurons to the electrical stimulation. The reverse-correlation approach, widely used to predict neural response to light stimulation, was adapted to predict neural response of ganglion cells to electrical stimulation. In contrast to the traditional reverse-correlation schemes, the model proposed here incorporated the history of the response of a neuron. To emphasize this, we call it the “spike history” model. To estimate the responses of cells to electrical stimulation, we used a novel pseudo-random stimulation. To validate the fitness of the model, we performed statistical analysis of the simulated spike trains. In particular, we compared the values of the coefficient of variation of the interspike interval for the experimentally recorded spike train, for the simulated spike train with the history model, and for the simulated spike train using the model without the response kernel. To compare how well the simulated spike train approximated experimentally recorded spikes, we compared the penalty terms for the spike history model and for the model without the response kernel. The penalty term was calculated based on the time difference between each simulated spike and the closest spike in time in the experimentally recorded train.

Cells responded to all stimulation frequencies. A phenomena of clusters of spikes followed by periods of suppression was observed in raw spike trains above 200 Hz stimulation. The phenomenon of cluster-suppression with white-noise stimulation may be indicative of memory in the system; i.e., the response depends not only on the current stimulus but also on the responses within a time window preceding the current time. To confirm this, we calculated the auto-correlation function of the recorded spike trains for different frequencies of stimulation. This showed that there was memory in the system for stimulation at frequencies higher than 100Hz. Robustness of the model parameters was confirmed by repeated stimulation of the same cell at the same frequency (up to 20 repetitions). Statistical comparison of the model with history and the model without history confirmed that the model with history approximates experimental data better.

The proposed model can be used to design a stimulation strategy to control neural excitation.

